# Draft Genome Sequence of an International Clonal Lineage 1 *Acinetobacter baumannii* Strain from Argentina

**DOI:** 10.1128/genomeA.01190-14

**Published:** 2014-11-26

**Authors:** Elisabet Vilacoba, Maxime Déraspe, German M. Traglia, Paul H. Roy, María Soledad Ramírez, Daniela Centrón

**Affiliations:** a Instituto de Microbiología y Parasitología Médica (IMPaM, UBA-CONICET), Facultad de Medicina, Universidad de Buenos Aires, Argentina; b Département de Biochimie, de Microbiologie, et de Bio-informatique and Centre de Recherche en Infectiologie, Université Laval, Québec, Canada; c Center for Applied Biotechnology Studies, Department of Biological Science, California State University, Fullerton, California, USA

## Abstract

In the last few years *Acinetobacter baumannii* has emerged worldwide as an important nosocomial pathogen in medical institutions. Here, we present the draft genome sequence of the international clonal lineage 1 (ICL1) *A. baumannii* strain A144 that was isolated in a hospital in Buenos Aires City in the year 1997. The strain is susceptible to carbapenems and resistant to trimethoprim and gentamicin.

## GENOME ANNOUNCEMENT

*Acinetobacter baumannii* often causes infections in hospitalized patients, being one of the major causes of ventilator-associated pneumonia (1–3).

Extreme levels of resistance, including resistance to carbapenems, tigecycline, and also colistin, has dramatically increased in the past years (4, 5). In Argentina, *A. baumannii* has been a worrisome problem since the late 1990s, when numerous outbreaks caused by this microorganism occurred in clinical settings, in particular in intensive care units (6).

To date, there are only two whole-genome sequences of *A. baumannii* strains isolated in Argentina available in GenBank. One of them corresponds to the naturally competent *A. baumannii* strain A118 and the other to a recently extensively drug-resistant *A. baumannii* indigo-pigmented strain A33405 (7, 8). Here, we announce the draft genome sequences of *A. baumannii* 144, which was isolated in 1997, when carbapenems were introduced into our clinical settings.

The multilocus sequence typing technique classified *A. baumannii* 144 as international clonal lineage 1 (ICL1), which is not the predominant clonal lineage disseminated in Argentina (9).

Whole-genome shotgun sequencing was performed using Illumina MiSeq- I, using Nextera XT libraries for sample preparation. Reads were assembled with the Ray assembler (http://denovoassembler.sourceforge.net).

The draft genome sequence of A144 consists of 92 contigs (length >500 bp), a total sequence of 4,312,914 bp with an *N*_50_ contig size of 89,819. The GC% average was 39.2. Using the RAST server to detect coding regions, we identified 4,151 possible ORFs. There are six rRNA operons and 69 tRNAs.

A more exhaustive analysis of the drug-resistance determinants harbored by the A144 strain and their contexts and locations will be included in a future publication. The possibility of analyzing the complete genome sequence of this strain could shed more light on the knowledge of this important nosocomial pathogen. These results expand our understanding of the global public health problem caused by this pathogen and also allow us to compare the genome content of this strain isolated in an Argentinian hospital to those obtained in the rest of the world.

### Nucleotide sequence accession numbers.

This whole-genome shotgun project has been deposited at DDBJ/EMBL/GenBank under the accession number JQSF00000000. The version described in this paper is version JQSF01000000.
